# Annual Meeting of the National Veterinary and Sanitary Association of the United States

**Published:** 1887-01

**Authors:** 


### PROCEEDINGS VETERINARY MEDICAL SOCIETIES.

### Annual Meeting of the National Veterinary and Sanitary Association of the United States.

President Huidekoper in the chair. The committee of investigation submitted the following report:

Your committee on investigation have had under consideration the work assigned, and beg leave to report that the disease now prevalent among the cattle in the distillery stables and elsewhere in the city of Chicago and vicinity is contagious pleuro-pneumonia, lung plague, or lungen suche, which terms are synonomous. And your committee are further of the opinion that the only way in which the infected localities can be freed from the contagion is by the slaughter of all diseased and exposed animals and a careful disinfection of the infected premises.

The report was adopted. Dr. Hopkins introduced a resolution against inoculation, which was referred to a committee.

The report of the committee on permanent organization was read by Dr. Hopkins, of Wyoming. It was taken up section by section. After undergoing some modifications a constitution was adopted, as follows:

ARTICLE 1. This association shall be known as the National Veterinary and Sanitary Association of the United States. It shall consist of State, territorial and government veterinarians, members of sanitary boards and live stock commissions, and representatives of veterinary colleges, associations and journals.

ART. 2. The purpose of this association shall be to contribute to the diffusion of true science, particularly the knowledge of sanitary science, as applied to the prevention of the spread of contagious diseases among domestic animals.

ART. 3. The officers of this association shall consist of a president, two vice-presidents, a secretary and assistant secretary; all of whom shall be elected at each annual meeting, and a majority of all votes present shall be necessary to a choice.

ART. 4. It shall be the duty of the president to preside at all meetings of the association. The usual parliamentary rules shall govern.

ART. 5. It shall be the duty of the secretary to keep a correct record of the proceedings.

ART. 6. It shall be the duty of the president to appoint a committee at each annual meeting to take up a collection or make an assessment to defray necessary expenses.

The committee on resolutions reported the following:

*Whereas*, The existence of contagious pleuro-pneumonia among animals in the United States is annually a source of great loss.

*Whereas*, The great cattle industry, commerce and food supply of the country are peculiarly threatened by the recent extension of contagious pleuro-pneumonia, and

*Whereas*, Real and alleged outbreaks of contagious disease require constant investigation and control; therefore, be it

*Resolved*, That the State veterinarians and veterinary sanitary boards of the United States in convention assembled, recognize the wisdom of congress in establishing the bureau of animal industry, and while heartily appreciating the valuable services rendered by the bureau in the past, we would recommend such additional legislation as to enable it to effectually extirpate contagious pleuro-pneumonia, and to control such other diseases as may from time to time appear.

Dr. Hopkins said he must take exception to the allusion to the valuable services of the bureau of animal industry. This bureau had actually been useless. It possessed no power to go into any herd except by the courtesy of the owner.

Dr. Atkins and others supported the resolutions.

The association then proceeded to an election of permanent officers. J. L. Brush, of Colorado, was unanimously elected president. Dr. J. D. Hopkins, territorial veterinarian of Wyoming, and Dr. V. T. Atkinson, State veterinarian of Wisconsin, were elected first and second vice-presidents, respectively, without any opposition. Dr. Paul Paquin, State veterinarian, of Missouri, was elected secretary, and Dr. M. R. Trumbower, of Illinois, assistant secretary.

In taking the chair, Mr. Brush said he was particularly grateful to the delegates for electing him, because it was a recognition of the lay element. He hoped the association would be able to stamp out pleuro-pneumonia, for if it ever got a start on the ranges of Colorado and the West, it would never be stamped out, and it would annihilate the cattle interest there.

Dr. Hopkins asked if the committee on resolutions had no other resolutions to report. He had introduced at the morning session a set of resolutions against inoculation, and he would ask if the committee did not intend to report them back.

Dr. Atkinson said the committee had considered the resolutions and would beg leave to report them back to the board without recommendation. Dr. Hopkins moved the passage of the resolutions. Dr. Huidekoper gave a history of the practice of inoculation in European countries since it was first discovered. He thought inoculation had always been condemned except in localities already thoroughly infected. In this country, where the disease was not permanent, it would be highly dangerous. Dr. Atkinson said the committee had been in full accord with the sentiment of the resolutions, but had not wished to incumber the business of the association with these resolutions. It was the intention to present the resolutions adopted at this convention to the Cattle-Growers' Association, and if these resolutions were added to those urging the necessity of stamping out contagious pleuro-pneumonia, the effect of the latter would be weakened.

Dr. Faville said the question of inoculation was a technical one, and he did not want to see any side issues draw away the attention of the cattle-growers' convention from the vital issue.

The resolution was finally adopted. The president was instructed to present the resolution on the subject of pleuro-pneumonia and those of Dr. Hopkins at the cattle-growers' convention.

